# Genomic Insights into (Extreme) Life at the Bottom of the Sea

**DOI:** 10.1371/journal.pbio.0040425

**Published:** 2006-11-14

**Authors:** Liza Gross

If you lived some 2,500 meters below the ocean’s surface in waters oscillating between 2 ° and 40 °C, what sorts of genes would you need? In a new study, Kathleen Scott, Stefan Sievert, and their colleagues shed light on the special adaptations required for such extreme living by sequencing and analyzing the complete genome of the extremophilic bacterium *Thiomicrospira crunogena* XCL-2. First isolated in 1985 from deep-sea hydrothermal vents along the East Pacific Rise in the South Pacific, *T. crunogena* has since been found in both Atlantic and Pacific Ocean vents, revealing its critical role in these ecosystems. It belongs to the diverse group of bacteria called gammaproteobacteria, which includes the human pathogens *Escherichia coli* and *Salmonella*.

*T. crunogena* is what’s known as an obligate chemolithoautotroph—it can grow using carbon dioxide as its sole carbon source and (in this case) sulfur as an energy source. Much like photosynthetic bacteria and plants use the sun’s energy to produce food, *T. crunogena* uses the oxidation of reduced sulfur compounds as an energy source for carbon fixation (synthesizing carbon-based sugars and other molecules) and cellular maintenance. And like their photosynthetic counterparts, these chemolithoautotrophs function as primary producers at the base of their vent community.

Hydrothermal vents release geothermally heated seawater through fissures along the volcanically active mid-ocean ridge. These carbon dioxide- and sulfide-rich plumes periodically mix with cold, oxygenated bottom water, creating eddies and forcing hydrothermal vent communities to contend with dramatic fluctuations in environmental conditions. One way *T. crunogena* copes with these oscillations is by using carbon-concentrating mechanisms that allow growth to continue when carbon dioxide levels drop. Scott et al. studied the content and structure of the microbe’s genome for evidence of other adaptations required to thrive in its extreme environment.

The *T. crunogena* genome is confined to a single chromosome that is densely packed with genes involved in electron transport (used to gain energy from sulfur compounds), energy and carbon metabolism, along with those required for nucleotide and amino acid synthesis and other cellular processes. The authors found only one genetic system for energy generation (and a possible alternate), which is perhaps not surprising for a microbe with limited energy options. They found all the components of the Sox system, a sulfur-oxidizing pathway typically found in microbes with more flexible strategies for carbon and energy metabolism. Together, these Sox genes completely oxidize, or strip electrons, from a variety of reduced sulfur-related compounds (producing sulfate). The microbe also harbors an enzyme that stops short of complete oxidation to sulfate (producing elemental sulfur instead). Because complete oxidation generates enough acid to inhibit growth, the authors suspect that this alternate pathway may curtail further acidification when acid levels rise.

**Figure:**
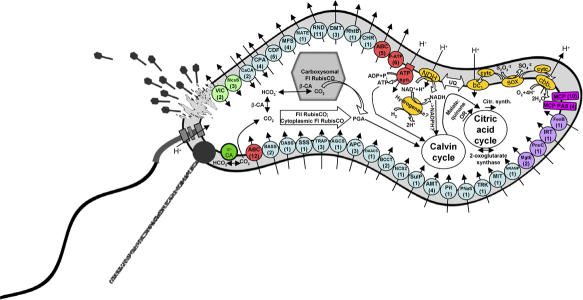
A model for metabolic function, based on the genome of *Thiomicrospira crunogena* XCL-2.

In any case, *T. crunogena* appears to be the first obligate chemolithoautotrophic sulfur-oxidizing bacteria to rely primarily on the Sox system for oxidizing sulfur compounds, raising the possibility that either the Sox system evolved in *T. crunogena*’s obligate autotrophic ancestors or the microbe lost its flexibility but retained its Sox pathway. Interestingly, however, this obligate chemotroph, which spends its life affixed to a hydrothermal vent, has proportionally more regulatory and signaling molecules than a free-living planktonic species. This enhanced repertoire likely reflects the different demands of life in extreme, volatile conditions—which requires rapid, flexible cellular responses—compared with the relatively stable existence of plankton floating on the open ocean.

*T. crunogena* also has more flexibility in acquiring nutrients, based on the number of genes encoding transporters involved in the uptake of inorganic nitrogen and phosphate. And the microbe has a number of chemotaxis genes, which likely direct it to favorable microhabitats along the vent, as well as pilin genes, which allow it to set up shop in the best locations.

With the first complete genome of a cosmopolitan autotrophic hydrothermal vent bacterium, researchers can further explore the genetic and physiological mechanisms that allow life to thrive in hostile environments at the bottom of the sea. And by comparing the *T. crunogena* genome to the genomes of autotrophic bacteria living in hot springs and other extreme environments around the world, they can begin to piece together the evolutionary history of primary production at the boundaries of life.

